# Transparent conductive oxide films mixed with gallium oxide nanoparticle/single-walled carbon nanotube layer for deep ultraviolet light-emitting diodes

**DOI:** 10.1186/1556-276X-8-507

**Published:** 2013-12-02

**Authors:** Kyoeng Heon Kim, Ho-Myoung An, Hee-Dong Kim, Tae Geun Kim

**Affiliations:** 1 School of Electrical Engineering, Korea University, Seoul 136-713, Korea; 2 Department of Digital Electronics, Osan College, Osan-si 447-749, Korea

**Keywords:** Gallium oxide (Ga_2_O_3_) nanoparticles (NPs), Single-walled carbon nanotubes (SWNTs), Ultraviolet transparent conductive oxide (UV TCO)

## Abstract

We propose a transparent conductive oxide electrode scheme of gallium oxide nanoparticle mixed with a single-walled carbon nanotube (Ga_2_O_3_ NP/SWNT) layer for deep ultraviolet light-emitting diodes using spin and dipping methods. We investigated the electrical, optical and morphological properties of the Ga_2_O_3_ NP/SWNT layers by increasing the thickness of SWNTs via multiple dipping processes. Compared with the undoped Ga_2_O_3_ films (current level 9.9 × 10^-9^ A @ 1 V, transmittance 68% @ 280 nm), the current level flowing in the Ga_2_O_3_ NP/SWNT increased by approximately 4 × 10^5^ times and the transmittance improved by 9% after 15 times dip-coating (current level 4 × 10^-4^ A at 1 V; transmittance 77.0% at 280 nm). These improvements result from both native high transparency of Ga_2_O_3_ NPs and high conductivity and effective current spreading of SWNTs.

## Background

High-brightness deep ultraviolet light-emitting diodes (UV LEDs) have attracted much attention in areas of air/water sterilization and decontamination, bioagent detection and natural light, identification, UV curing, and biomedical and analytical instrumentation [1]. To date, the maximum external quantum efficiency (EQE) for commercialization of deep UV LEDs is 3% at the wavelength of 280 nm [2,3]. Various reasons can account for the poor EQE, mainly such as relatively low-resistance ohmic contacts, low hole concentration in p-type AlGaN layer, and the absence of transparent conductive oxides (TCOs) electrode in the deep UV wavelength region [4,5]. In particular, it is believed that the development of high-performance TCOs electrode in the deep UV region is a key to increase the EQE of UV LEDs. Conventionally, indium tin oxide (ITO), which exhibits high conductance and good transparency in a visible region, has been widely used as the TCOs electrodes in LEDs and solar cells [6,7]. However, it has an opaque property in the deep UV (<300 nm) region due to a small bandgap (approximately 3.2 eV), and hence, new TCO materials need to be explored for deep UV LEDs. The wide bandgap materials such as SiO_2_, Si_3_N_4_, HfO_2_ are attractive as TCOs for deep UV LEDs because of their high transmittance in deep UV regions, but it is difficult to provide electrical conductivity into these materials. In the meantime, the gallium oxide with β phase (β-Ga_2_O_3_) having a large optical bandgap of 4.9 eV has been reported as a deep-UV TCO material [8] because its conductivity can be improved by thermal annealing, impurity doping, or incorporating some conducting paths using SWNTs. The Ga_2_O_3_ film has also excellent adhesion to GaN surfaces [9]. For example, since undoped Ga_2_O_3_ film has insulating properties (i.e., conductivity (*σ*) <10^-9^ Ω^-1^ · Cm^-1^), it was doped with tin (Sn) atoms to increase the conductivity at the expense of optical transmittance. For 3 mol% Sn-doped Ga_2_O_3_ films, the conductivity was increased up to 375 Ω^-1^ · Cm^-1^ (42 Ω/square) but the transmittance decreased to approximately 15% in the deep UV region (280 nm) [10]. In order to improve the low optical properties, several groups have reported synthesized TCO layer by wet-based nanoparticles (NPs), such as ITO, indium zinc oxide (IZO), antimony zinc oxide (AZO), antimony tin oxide (ATO), etc. [11-14]. This small particle size (i.e., NPs size), typically <30 nm, guarantees a low light scattering and thus allows a high optical quality of the materials [15]. Unfortunately, even with some improvement of optical properties, these synthesized TCO NP layers still do not satisfy the requirement for deep UV applications due to the added dopants such as Sn, Sb, In, Ga, etc. [16].

In this work, we propose a TCO electrode scheme of gallium oxide nanoparticle/single-walled carbon nanotube (Ga_2_O_3_ NP/SWNT) layer, consisting of undoped Ga_2_O_3_ NPs for high transmittance and SWNT for high conductivity, for deep UV LED applications.

## Methods

**Figure 1:**
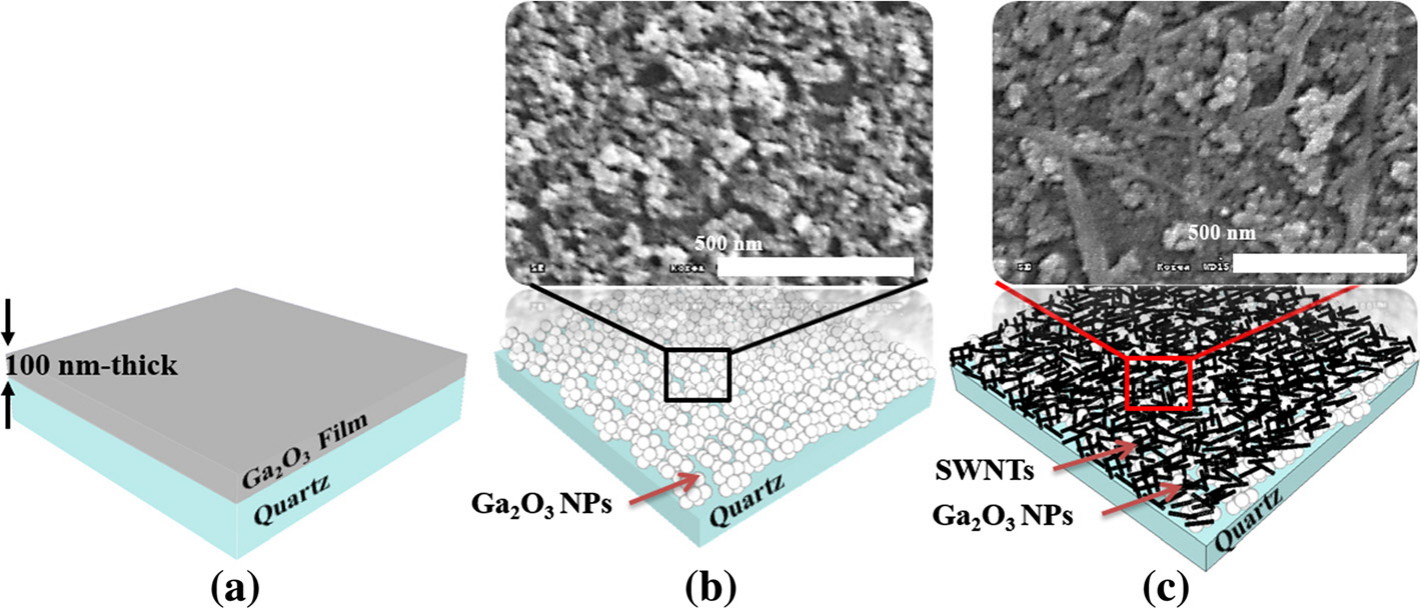
**Schematic illustration of the three samples. (a)** As-deposited undoped Ga_2_O_3_ film, **(b)** coated with undoped Ga_2_O_3_ NP layer, **(c)** combined with SWNT and Ga_2_O_3_ NP layer on quartzs.

In order to directly compare the optical and electrical properties, three samples - i.e. as-deposited undoped Ga_2_O_3_ films, coated with undoped Ga_2_O_3_ NP layers, and combined with SWNTs and Ga_2_O_3_ NP layer - were prepared on quartzs, as depicted in Figure 1. First, undoped Ga_2_O_3_ films were deposited on normal quartz substrates by radio frequency (RF) magnetron sputtering of Ga_2_O_3_ ceramic targets (purity of 99.99%), as shown in as a Figure 1a. The sputtering chamber was pumped down to 2 × 10^-6^ before introducing argon gas. The sputtering was carried out under a pressure of 5 mTorr in pure argon atmosphere. The film was then grown at room temperature with a target RF power of 100 W, and the thicknesses of undoped Ga_2_O_3_ layer, determined by Alpha step profilometer, were about 100 nm. Second, it is a prerequisite to achieve the uniform coating of Ga_2_O_3_ NP layers prior to the fabrication of the proposed Ga_2_O_3_ NP/SWNT layer. Only undoped Ga_2_O_3_ NP layer with sizes less than 15-nm diameter for high transmittance was coated by simple spin-coating methods, as shown in Figure 1b. Finally, in order to combine the undoped Ga_2_O_3_ NP layer on quartz and the SWNTs for high conductivity, SWNT solution (0.5 mg/ml) with sizes less than 7 μm length in dichlorobenzene (DCB) was dispersed using the ultrasonic for 24 h, as shown in Figure 1c. The Ga_2_O_3_ NPs coated in a single layer can increase the adhesion of SWNTs on the substrate [9], eventually leading to more uniform and stable TCO films.

**Figure 2:**
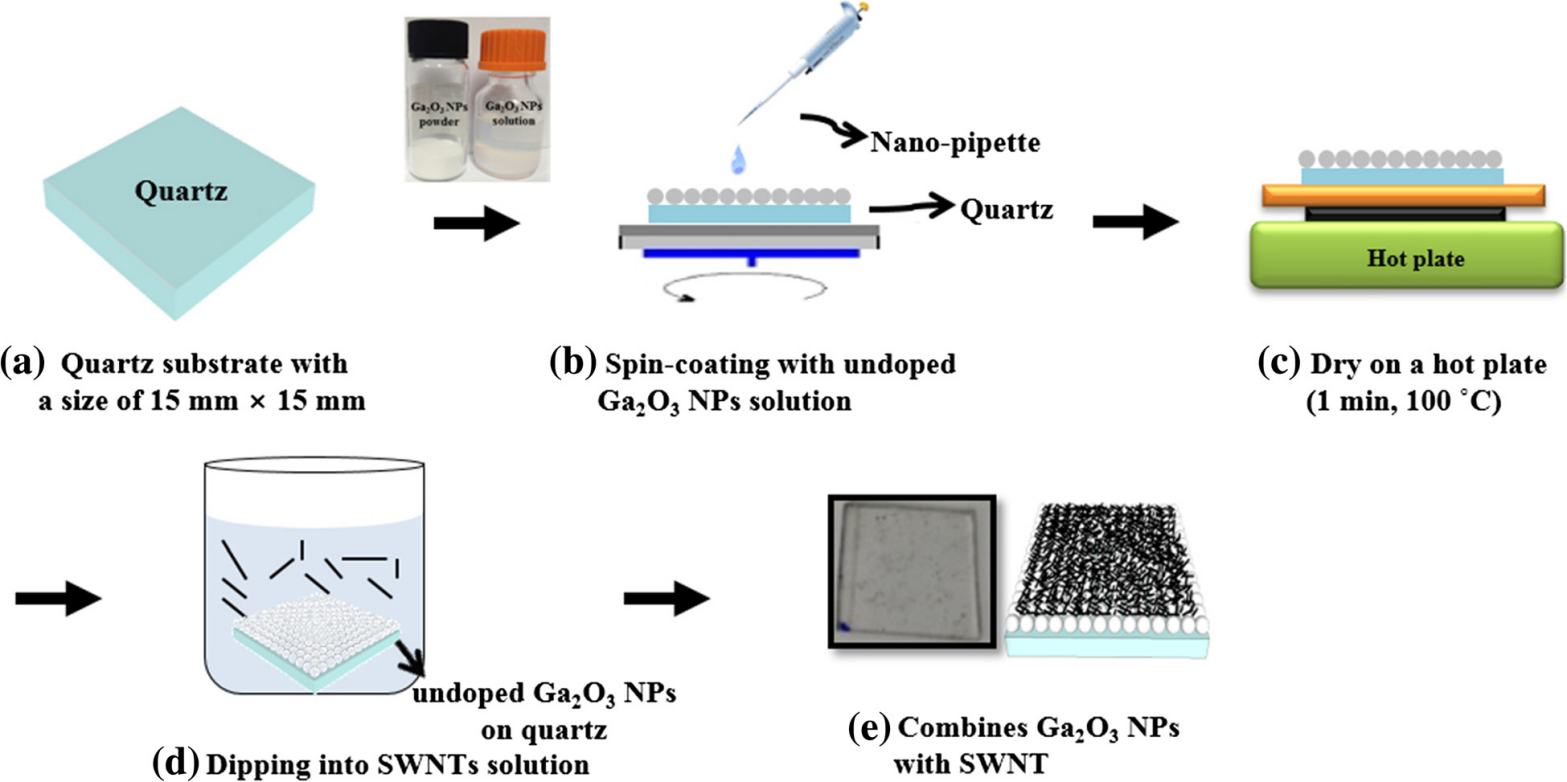
**The schematic illustration for spin and dip-coating procedure of proposed Ga**_**2**_**O**_**3 **_**NP/SWNT layer on quartzs.**

Figure 2 shows the schematic illustration of the spin and dip-coating procedure of the proposed Ga_2_O_3_ NP/SWNT layer on quartz. All the quartz substrates with a size of 15 mm × 15 mm were ultrasonically cleaned and dried in flowing nitrogen gas, as shown in Figure 2a. And then, in order to make the substrates hydrophilic, the substrates are sonicated for 1 h in RCA (5:1:1, H_2_O/NH_4_OH/30% H_2_O_2_) solution, which adds many -OH groups to the surface [17]. Continuously, in order to prepare the undoped Ga_2_O_3_ NP solution with a concentration of 60 wt.%, 30 mg of undoped Ga_2_O_3_ nanopowder with an average size of 15 nm were mixed with 20 ml of ethanol and sonicated overnight. And then, the ready solution was coated on quartz substrates using the spin-coating technique, as shown in Figure 2b. After spin coating, each sample was dried on a hot plate for 1 min at 100°C, as shown in Figure 2c. Here, this sequential step in Figure 2a,b,c is defined as a 'one cycle’ of coated undoped Ga_2_O_3_ NP layer on the substrate. This cycle was controlled by spin-coating process parameters, such as the solution concentration of undoped Ga_2_O_3_ NPs, coating velocity and time, and cycle number, for uniform surface with undoped Ga_2_O_3_ NP layer on the quartz. Finally, in order to combine the undoped Ga_2_O_3_ NP layer on quartz and the SWNTs for high conductivity, SWNT solution (0.5 mg/ml) in DCB was dispersed using the ultrasonic for 24 h. And then, the substrate coated the undoped Ga_2_O_3_ NP layer was dipped in a SWNT solution for 3 min and dried in flowing nitrogen gas, as shown in Figure 2d. Both the schematic and corresponding optical image for the SWNTs/Ga_2_O_3_ NP layer are shown in Figure 2e.

The surface morphology of the films was observed by a scanning electron microscope (SEM, Hitachi S-4300, Tokyo, Japan). In order to confirm the electrical properties, the sheet resistance and current-voltage (*I-V*) characteristics of the Ga_2_O_3_ NP/SWNT layer were measured by four-point probe method (CMT-SR1000N digital four-point testing instrument, AIT, Korea) and the semiconductor parameter analyzer (Keithley 4200-SCS, Tokyo, Japan), respectively. The optical transmission was measured using a double beam spectrophotometer (PerkinElmer, Lambda 35, Waltham, MA, USA) in the wavelength range of 280 to 700 nm.

## Results and discussion

**Figure 3:**
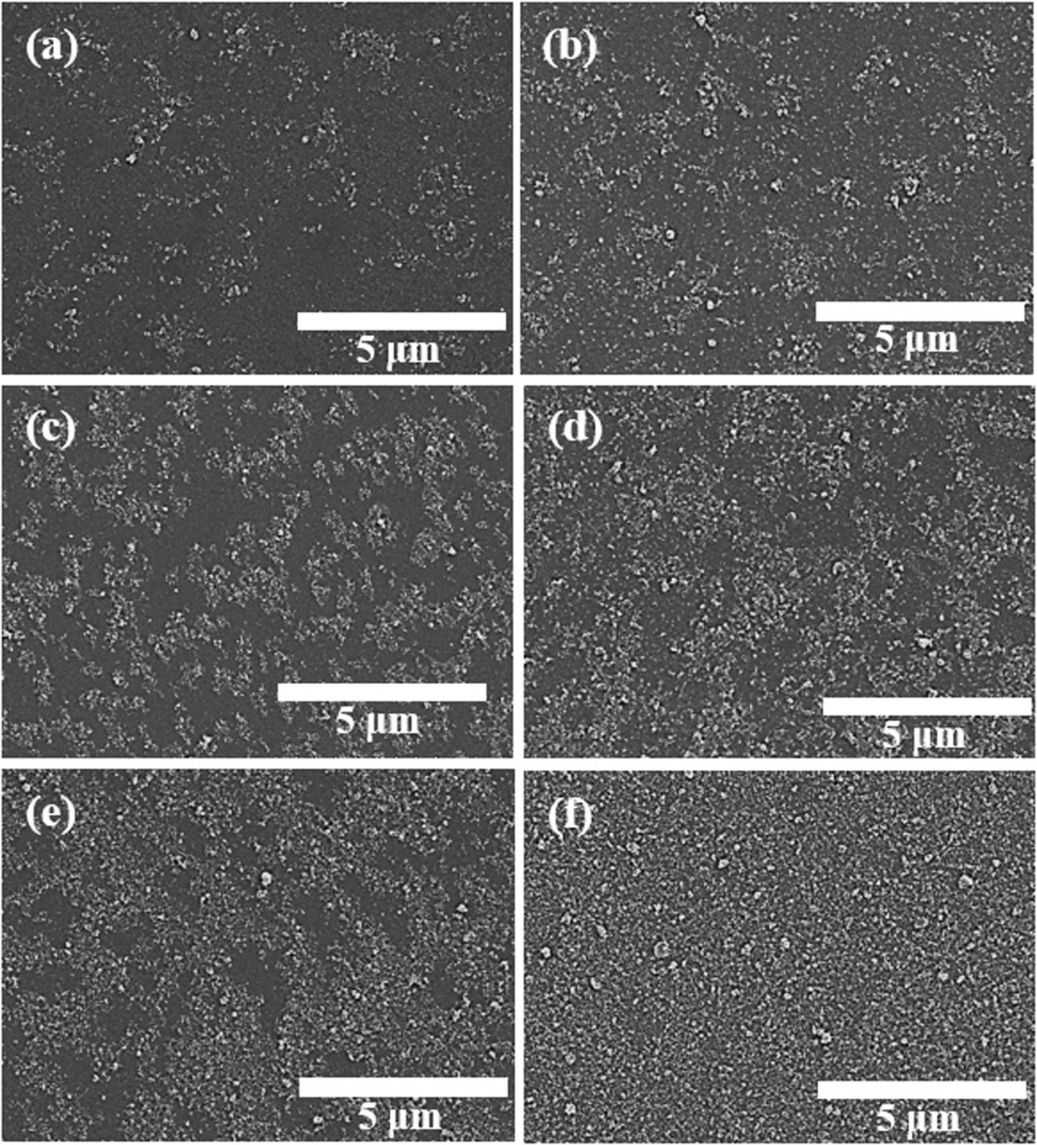
**SEM images of undoped Ga**_**2**_**O**_**3 **_**NP layer coated under different coating cycles on quartzs. (a)** 1 cycle, **(b)** 2 cycles, **(c)** 3 cycles, **(d)** 4 cycles, **(e)** 5 cycles, **(f)** 6 cycles.

In order to realize our proposed scheme, the uniform coating conditions of the undoped Ga_2_O_3_ NP layer should be preceded by using the spin-coating method. Figure 3 shows the SEM image of undoped Ga_2_O_3_ NP layer coated in different coating cycles on quartzs. The undoped Ga_2_O_3_ NP layer coated by one cycle was remained roughly uniform on the macro-scale, as shown in Figure 3a. The uniform formation of the undoped Ga_2_O_3_ NP layer is associated with wettability of the quartz substrate. If the substrate wets nicely with the spin-coating solvent, the undoped Ga_2_O_3_ NP layer could extend quickly on the substrate and the solvent rapidly evaporated at the same time. The undoped Ga_2_O_3_ NPs were then gradually aggregated in a microscale size as the number of coating cycles increased.

Consequently, we obtained the most uniform condition after the 6-cycle repetitive coating, as shown in Figure 3f.

**Figure 4:**
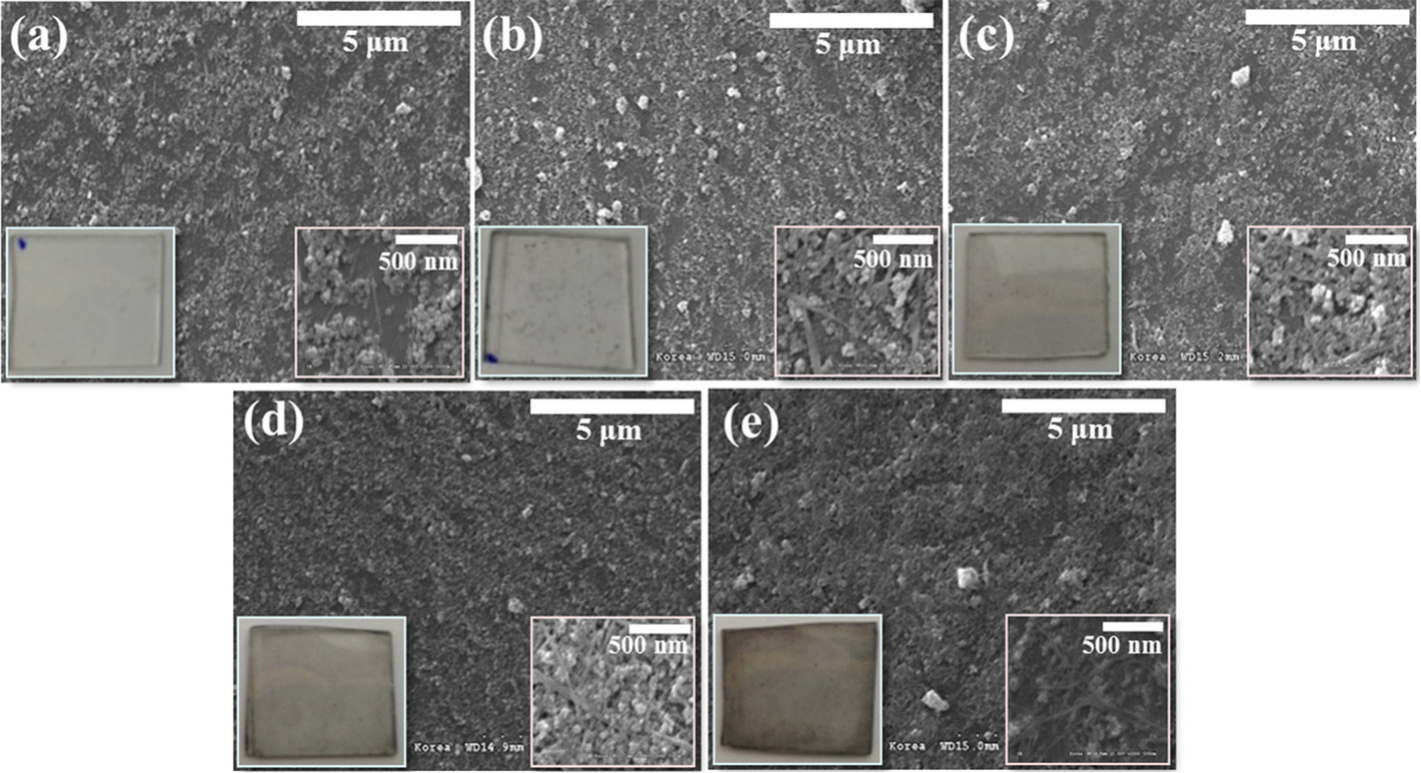
**SEM images and photographs of combined Ga**_**2**_**O**_**3 **_**NP/SWNT layers under different SWNT solution dipping times on quartz. (a)** 5 times, **(b)** 10 times, **(c)** 15 times, **(d)** 20 times, **(e)** 25 times.

Figure 4 shows the SEM surface images of the combined Ga_2_O_3_ NP/SWNT layer, under different SWNT solution dipping times. The undoped Ga_2_O_3_ NP layers optimized from the SEM data in Figure 3 were used in this experiment. With increasing the number of dipping times, the number of intersection points in the SWNT network considerably increased, providing effective conducting pathways; this increased pathway in the network structures on the undoped Ga_2_O_3_ NP layer may enhance the electrical conductivity of the Ga_2_O_3_ NP/SWNT layer [18,19]. However, if the number of dip-coating of the SWNT solution is more than 20 times, the optical transmittance would be decreased due to the increase of dark areas by the SWNT network, as shown in Figure 4d.

**Figure 5:**
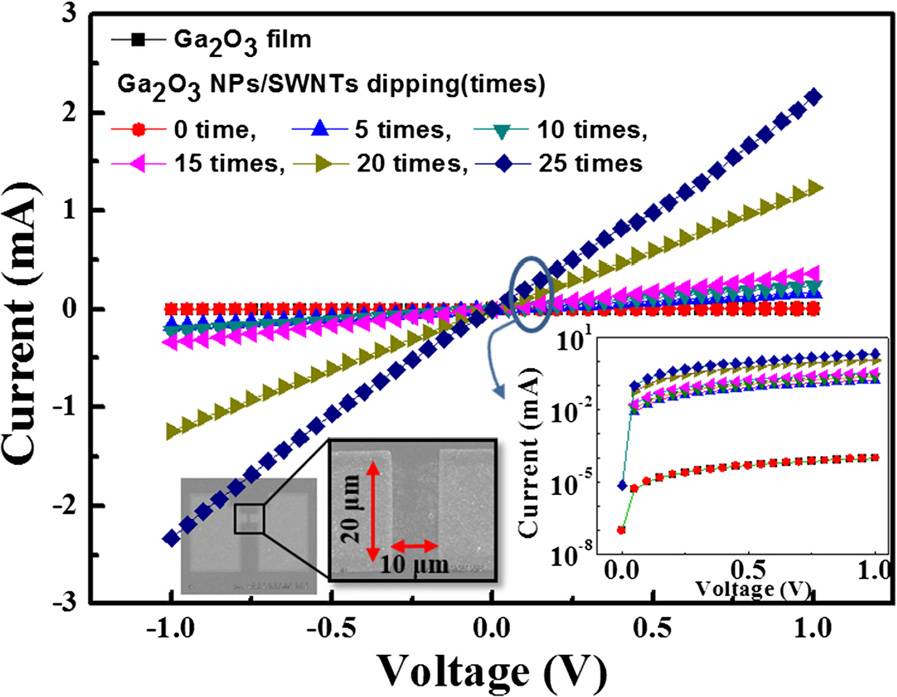
**Current-voltage characteristic curves.** Measured for samples bridged over aluminum (Al) metal pads on p-type Si wafer with *n*-doped Ga_2_O_3_ film, Ga_2_O_3_ NP layer, and Ga_2_O_3_ NP/SWNT layer obtained by varying the dipping times in SWNT-dispersed solution (Inset: SEM images of the channel bridged with various films between the two Al metal pads formed on p-type Si wafer with a size of 10 μm × 20 μm).

Then, we investigated the electrical and optical properties according to the SWNT adsorption, as shown in Figure 5. Figure 5 shows the *I-V* curve characteristics with sweep voltages ranging from -1 to 1 V for three samples (i.e., undoped Ga_2_O_3_ film, undoped Ga_2_O_3_ NP layer, and Ga_2_O_3_ NP/SWNT layer). For the characterization, the current electrode pad with a size of 10 μm × 20 μm was fabricated with Al metal electrodes on the SiO_2_ layer-grown p-type Si wafer using a photolithography process, as shown in the insets of Figure 5[20]. As a result, the current level of undoped Ga_2_O_3_ film and undoped Ga_2_O_3_ NP layer at 1 V were 99 and 98 nA, whereas the Ga_2_O_3_ NP/SWNT layer showed a significant increase of the current flows at 0.4 mA (at 1 V) for 15 times dipping. These results for the undoped Ga_2_O_3_ film and undoped Ga_2_O_3_ NP layer can be attributed to the intrinsically insulating property of Ga_2_O_3_ with a bandgap of 4.8 eV. Although the current significantly dropped in the presence of the undoped Ga_2_O_3_ NP layer owing to its high resistance, the Ga_2_O_3_ NP/SWNT layer exhibited high current level. These contrary *I-V* characteristics of undoped Ga_2_O_3_ NP layer and Ga_2_O_3_ NP/SWNT layer may result from the SWNT network of high conductivity [18]. This effective reduction in the resistance results from the formation of the principal conducting pathways by the increase in the bundle to bundle junction, as shown in Figure 4. These conducting pathways are related to the contact area of undoped Ga_2_O_3_ NP layer substrate [21]. Compared with the conventional film, undoped Ga_2_O_3_ NP layer may have a larger contact cross-sectional area, leading to lower resistance.

**Figure 6:**
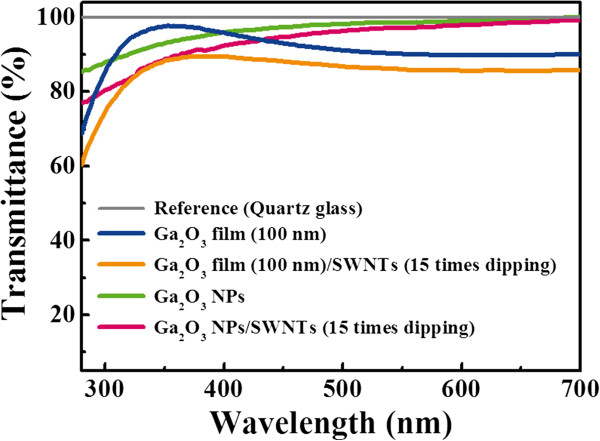
**Optical transmittance spectra of undoped Ga_2_O_3_ film, Ga_2_O_3_ NP layer, and Ga_2_O_3_ NP/SWNT layer deposited on quartz.** Under 15 times of dipping in SWNT-dispersed solution.

Figure 6 shows the transmittance spectra of the four samples. Transmittance of undoped Ga_2_O_3_ film, Ga_2_O_3_/SWNT film, the undoped Ga_2_O_3_ NP layer, and Ga_2_O_3_ NP/SWNT layer were to be 68.6%, 60.4%, 85.4%, and 77.0% at a wavelength of 280 nm, respectively. Compared with the undoped Ga_2_O_3_ film, the undoped Ga_2_O_3_ NP layer shows approximately 17% higher optical transmittance. This improvement may be attributed to the reduced optical light scattering via undoped Ga_2_O_3_ NPs (<15 nm in diameter). On the other hand, the transmittance was decreased by 8.4% due to the optical loss by SWNTs after one dipping; however, it is still good enough to use in the deep UV region as well as visible region [22]. By comparison, the transmittances of oxide-based TCOs were reported to be lower than 40% at 280 nm [23,24] while those of the immersing electrodes such as SWNT, graphene, and Ag nanowire thin films were approximately 70% at 280 nm [25].

**Figure 7:**
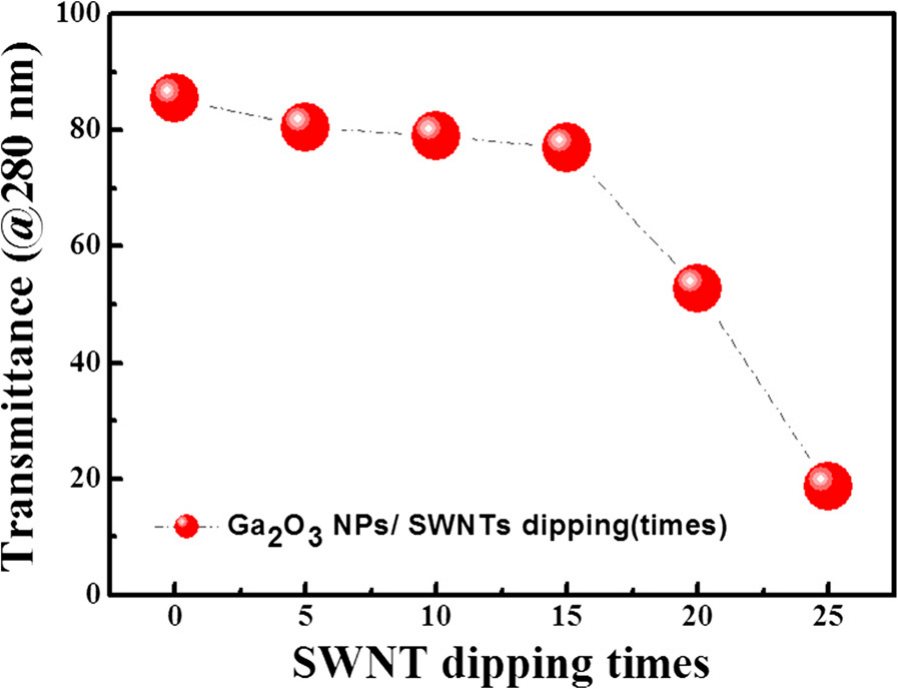
Optical transmittance versus SWNT solution dipping times measured for the Ga_2_O_3_ NP/SWNT layer.

In order to determine the optimal transmittance for SWNT solution dipping times, Figure 7 shows the relationship between the transmittance at 280 nm and SWNT solution dipping times. The optical transmittance is reduced with increasing the dipping times. That is, the transmittance values were 85.4%, 80.5%, 79.0%, 77.0%, 52.7%, and 18.6% after dipping treatments of 0, 5, 10, 15, 20, and 25 times, respectively. The reduction ratio of the transmittance is not so great (5% to 8%) for 0 to 15 dipping time ranges. For example, 15 times of dipping samples show a slight decrease in the transmittance due to the coverage with SWNTs on the undoped Ga_2_O_3_ NP layer, but a remarkable influence on the reduction of the transmittance, whereas it provided pronounced enhancement effect in electrical conductivity, as shown in Figure 5. From these results, we can conclude that our proposed TCO scheme of the Ga_2_O_3_ NP/SWNT layer may be useful as an electrode for deep UV LEDs. However, the resistivity of Ga_2_O_3_ NP/SWNT layer is approximately 3 orders higher in magnitude than that observed for commercial ITO films [26], and should be further reduced by introducing doped Ga_2_O_3_ NPs without transmittance loss.

## Conclusions

We proposed and investigated the electrical and optical properties of undoped Ga_2_O_3_ NP layer combined with SWNTs by using the simple spin and dip-coating methods for deep UV LEDs. From the *I-V* curve characteristics, the Ga_2_O_3_ NP/SWNT layer showed a high current level of 0.4 × 10^-3^ A at 1 V. Compared with the undoped Ga_2_O_3_ NP layer, optical transmittance of Ga_2_O_3_ NPs/SWNT layer after 15 times of dipping was decreased by only 15% at 280 nm. By adjusting the dipping times in the Ga_2_O_3_ NP/SWNT layer, we obtained improved optical transmittance of 77.0% at 280 nm after 15 times of dip-coating processes.

## Competing interests

The authors declare that they have no competing interests.

## Authors’ contributions

KHK, HMA, and HDK performed all the research and carried out the analysis. TGK supervised the work and drafted the manuscript. TGK revised the manuscript critically and provided theoretical guidance. All authors read and approved the final manuscript.
